# Polystyrene nanoplastic exposure actives ferroptosis by oxidative stress-induced lipid peroxidation in porcine oocytes during maturation

**DOI:** 10.1186/s40104-024-01077-6

**Published:** 2024-09-03

**Authors:** Yijing He, Tianhang Yu, Heran Li, Qinfeng Sun, Miaoyu Chen, Yiyi Lin, Jianjun Dai, Weihan Wang, Qiao Li, Shiqiang Ju

**Affiliations:** 1 MOE Joint International Research Laboratory of Animal Health and Food Safety, College of Veterinary Medicine, Nanjing Agricultural University, Nanjing, 210095 China; 2 Key Laboratory of Livestock and Poultry Resources (Pig) Evaluation and Utilization, Ministry of Agriculture and Rural Affairs, Institute of Animal Husbandry and Veterinary Science, Shanghai Academy of Agricultural Sciences, Shanghai, 201106 China

**Keywords:** Ferroptosis, Lipid peroxidation, Mitochondria, Polystyrene nanoplastics, Porcine oocyte, ROS

## Abstract

**Background:**

Polystyrene nanoplastics (PS-NPs) are becoming increasingly prevalent in the environment with great advancements in plastic products, and their potential health hazard to animals has received much attention. Several studies have reported the toxicity of PS-NPs to various tissues and cells; however, there is a paucity of information about whether PS-NPs exposure can have toxic effects on mammalian oocytes, especially livestock. Herein, porcine oocytes were used as the model to investigate the potential effects of PS-NPs on mammalian oocytes.

**Results:**

The findings showed that different concentrations of PS-NPs (0, 25, 50 and 100 μg/mL) entering into porcine oocytes could induce mitochondrial stress, including a significant decrease in mitochondrial membrane potential (MMP), and the destruction of the balance of mitochondrial dynamic and micromorphology. Furthermore, there was a marked increase in reactive oxygen species (ROS), which led to oocyte lipid peroxidation (LPO). PS-NPs exposure induced abnormal intracellular iron overload, and subsequently increased the expression of transferrin receptor (TfRC), solute carrier family 7 member 11 (SLC7a11), and acyl-CoA synthetase long-chain family member 4 (ACSL4), which resulted in ferroptosis in oocytes. PS-NPs also induced oocyte maturation failure, cytoskeletal dysfunction and DNA damage. Cotreatment with 5 μmol/L ferrostatin-1 (Fer-1, an inhibitor of ferroptosis) alleviated the cellular toxicity associated with PS-NPs exposure during porcine oocyte maturation.

**Conclusions:**

In conclusion, PS-NPs caused ferroptosis in porcine oocytes by increasing oxidative stress and altering lipid metabolism, leading to the failure of oocyte maturation.

**Graphical Abstract:**

PS-NPs could enter oocytes, caused mitochondrial dysfunction and oxidative stress, induced lipid peroxidation and ferroptosis, which eventually resulted in failure of oocyte maturation.

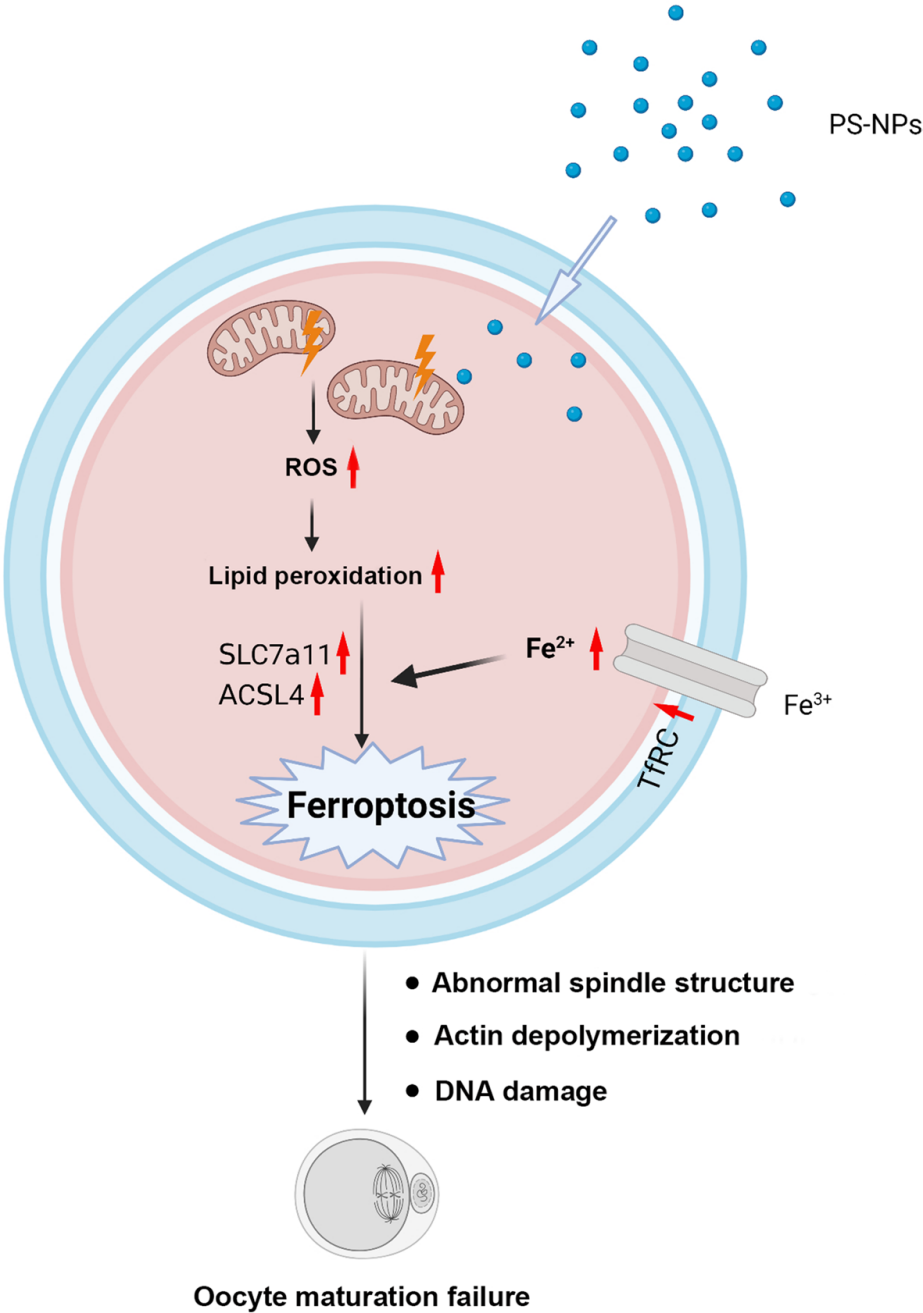

**Supplementary Information:**

The online version contains supplementary material available at 10.1186/s40104-024-01077-6.

## Introduction

Plastics have greatly improved the quality and efficiency of human life. However, the environmental pollution caused by plastic waste products is currently a major concern. Plastic litter in the environment gradually degrades into secondary microplastics (MPs) as a consequence of solar radiation, thermal oxidation, and wave action [1]. Based on the size of plastic residues, particles are divided into MPs and nanoplastics (NPs), MPs refer to plastic particles with a diameter of less than 5 mm and NPs with a diameter of less than 1 μm [2, 3]. MPs and NPs are extensively distributed in the environment, including soil, air and marine organisms, even in bottled drinking water [4–6]. Notably, polystyrene (PS) is widely applied in commercial products, yet the potential health risks of polystyrene nanoplastics (PS-NPs) have aroused widespread public concern. Several studies have shown that the continuous exposure of living organisms to PS-NPs can lead to PS-NPs aggregation in different organs, resulting in toxic effects, including antioxidant dysfunction, immune responses and inflammation [7–10].

An in vivo study reported that oral exposure to PS-NPs caused intestinal barrier dysfunction and increased intestinal permeability [11]. PS-NPs (≤ 0.1 μm) can pass through cell membranes and then interfere with cellular processes and interact with organelles [10]. After passing through the membrane, PS-NPs become stressors in various mammalian cells, increasing ROS levels, disrupting redox balance, and causing further antioxidant responses and autophagy [12]. Several studies have shown that the toxic effects of PS-NPs are related to mitochondrial dysfunction [13], endoplasmic reticulum stress [14], lysosomal stress [15] and DNA damage [16, 17].

Ferroptosis is a programmed death characterized by iron overload and abnormal polyunsaturated fatty acid (PUFA) metabolism [18]. ROS interact with PUFAs to form LPO products such as 4-hydroxynonenal (4-HNE) and malondialdehyde (MDA) [19]. The excessive accumulation of ROS is also associated with ferroptosis-dependent cell death. Ferroptosis is also defined as a mode of programmed death that relies on iron and ROS [18, 20]. It is now recognized from a number of studies that nanoparticles can disrupt iron transportation and induce ferroptosis in mammalian cells [18, 20]. A recent study revealed a strong link between PS-NPs and ferroptosis in the mouse intestine [21].

PS-NPs have been reported to trigger toxic effects in different organs and cells; however, little is known about whether PS-NPs have adverse effects on mammalian oocytes, especially in livestock species. Considering the substantial fatty acid reserves within cytoplasmic lipid droplets [22], we hypothesized that porcine oocytes might be susceptible to ROS attack and cause lipid peroxidation (LPO). Therefore, porcine oocytes were used as the model in this study to investigate the potentially toxic effects of PS-NPs on oocytes through oxidative stress, lipid metabolism and ferroptosis.

## Materials and methods

### Antibodies and chemicals

Phospho-histone H_2_A.X (S139) rabbit antibody was obtained from Abmart Shanghai Co., Ltd. (T56572, Shanghai, China). Anti-ACSL4 recombinant rabbit monoclonal antibody (ET7111-43), anti-SLC7a11 mouse monoclonal antibody (HA600098), anti-GPX4 recombinant rabbit monoclonal antibody (ET1706-45) and anti-TfRC recombinant rabbit monoclonal antibody (ET1702-06) were purchased from HUABIO (Hangzhou, China). The suspension of red fluorescent PS-NPs beads with a sizes of 100 nm (7-1-0010, solid content: 1%, solution) was obtained from Tianjin Baseline ChromTech Research Centre (Tianjin, China). The suspension of unmodified PS-NPs beads with size of 100 nm (PC-100, solid content: 5%, solution) was purchased from Janus New-Materials Co., Ltd. (Nanjing, China). FerroOrange (F374) and Liperfluo (L248) were purchased from Dojindo Molecular Technologies Inc. (Shanghai, China).

### Oocytes in vitro maturation (IVM) and PS-NPs exposure

Porcine ovaries were acquired from a local abattoir and transported to the laboratory in 37 °C sterile physiological saline. Cumulus oocyte complexes (COCs) were aspirated from antral follicles and transferred into pre-equilibration TCM-199 medium supplemented with 5.0 mg/mL streptomycin, 10% (v/v) porcine follicular fluid, 3.05 nmol/L D-glucose, 0.1% polyvinyl alcohol (w/v), 26.19 mmol/L NaHCO_3_, 0.91 mmol/L sodium pyruvate, 0.57 mmol/L L-cysteine, 10 IU/mL PMSG and 10 IU/mL hCG, 10 ng/mL EGF, for IVM [23]. Approximately 50 COCs in one well of four-well dish containing 500 μL of TCM-199 medium and covering 200 μL mineral oil. The COCs were cultured for 44 h at 38.5 °C in a humidified atmosphere with 5% CO_2_.

The unmodified PS-NPs beads and red fluorescent-labeled PS-NPs beads were diluted with TCM-199 IVM medium to concentrations of 25, 50 and 100 μg/mL for treatment. For ferrostatin-1 (Fer-1, SML0583, Sigma-Aldrich) treatment, a stock solution (10 mmol/L) of Fer-1 was prepared in DMSO and then diluted to 1, 5, 10 μmol/L with TCM-199 IVM medium before use. The COCs were randomly allocated to 50 μg/mL PS-NPs groups and 50 μg/mL PS-NPs + Fer-1 cotreatment group. Cumulus cells were removed by 0.1% (w/v) hyaluronidase after 44 h of culture, and then the denuded oocytes were collected for subsequent experiments.

### Immunofluorescences staining

After being fixed for 30 min with 4% paraformaldehyde and permeabilized for 8 h with immunostaining permeabilization buffer, the oocytes were blocked in immunostaining blocking buffer and then incubated with anti-γ-H_2_A X antibody (1:200) for 4 h at room temperature. The oocytes were transferred to the secondary antibody at 37 °C for 1 h. Finally, DNA was labeled with 10 mg/mL Hoechst 33342 for 15 min at 37 °C, and the oocytes were mounted in glycerol on the glass slides. The fluorescence was analyzed by a laser confocal scanning microscope (Zeiss LSM 700 META, Oberkochen, Germany), and then analyzed using the ImageJ software (National Institutes of Health, Bethesda, MD, United States). The mean gray value of each oocyte was equal to the integrated density divided by the area.

### RNA extraction and real-time quantitative PCR (qPCR)

Total RNA from 100 oocytes in each group was extracted by SteadyPure Universal RNA Extraction kit (AG21017, Accurate Biology, Changsha, China) and synthesized to cDNA. qPCR was conducted on a real-time PCR instrument (QuantStudio 6 Flex). Primer sequences were listed in Additional file 1. The data were analyzed using the 2^−ΔΔCt^ method.

### Measurement of mitochondrial membrane potential (MMP)

MMP in oocytes was evaluated by a mitochondrial membrane potential assay kit with JC-1 (C2006, Beyotime Biotechnology, Shanghai, China). The oocytes were transferred to pre-equilibrated working solution and incubated for 30 min at 37 °C. The fluorescent signals were examined by a laser confocal scanning microscope.

### Transmission electron microscopy (TEM)

Oocyte samples were fixed in 2.5% glutaraldehyde and 1.5% paraformaldehyde at 4 °C overnight and then fixed in osmium at 4 °C for 1 h. The samples were progressively dehydrated, replaced, dispersed and polymerized in a polymerization reactor. After trimming and re-embedding, the samples were stained with uranyl acetate-lead citrate. Samples were observed under transmission electron microscope (Hitachi 7800).

### ROS measurement

Reactive Oxygen Species Assay kit (S0033, Beyotime Biotechnology, Shanghai, China) was used to analyze the ROS levels in oocytes. The oocytes were incubated with 10 μmol/L DCFH-DA (Dichlorofluorescein diacetate) for 30 min at 37 °C and then the fluorescence was analyzed with a confocal fluorescent microscope.

### Malondialdehyde (MDA) assay

After PS-NPs exposure for 44 h, 350 oocytes from each group were collected and lysed in RIPA lysis buffer with 1 mmol/L phenylmethylsulfonyl (abs812852, Absin, Shanghai, China) on ice for 30 min. The MDA level was analyzed using MDA assay kit (S0131, Beyotime Biotechnology, Shanghai, China) according to the manufacturer’s instructions. The stock solution of thiobarbituric acid (TBA) with a concentration of 0.37%. The mixture of oocyte samples and TBA reagent was incubated at 100 °C for 15 min and the absorbance was detected at 532 nm.

### LPO imaging

To visualize LPO, the oocytes were incubated in 25 μmol/L Liperfluo for 30 min. The fluorescence signals of LPO were observed by a laser confocal scanning microscope.

### Iron assay

The intercellular Fe^2+^ levels were assessed using a fluorescent probe Ferro Orange. The oocytes were transferred to the 5 μmol/L fluorescent probe for 30 min at 37 °C. The fluorescence signals were observed by a laser confocal scanning microscope.

### Western blot analysis

After PS-NPs exposure for 44 h, oocytes in each group were collected and lysed in RIPA lysis buffer for 30 min. Samples were boiled at 100 °C for 10 min and stored at –80 °C. A total of 10 μg protein was separated and then transferred to PVDF membranes. The membranes were blocked by 5% skim milk and then incubated with primary antibody for 8 h at 4 °C, then, the membranes were incubated with HRP-labeled secondary antibodies for 1 h at 37 °C. Finally, proteins were visualized with ECL Chemiluminescent Substrate (BL520A, Bioshap Technology Co., Ltd., Anhui, China).

### Statistical analysis

Three replicates were conducted for each experiment. One-way ANOVA was used to evaluate the differences between groups using Graph Pad 8.0. The results are presented as mean ± standard error (SE) values. Differences of *P* < 0.05 were considered significant.

## Results

### PS-NPs exposure resulted in the first polar body (PB1) extrusion failure and cell cycle progression interruption in porcine oocytes

**Fig. 1:**
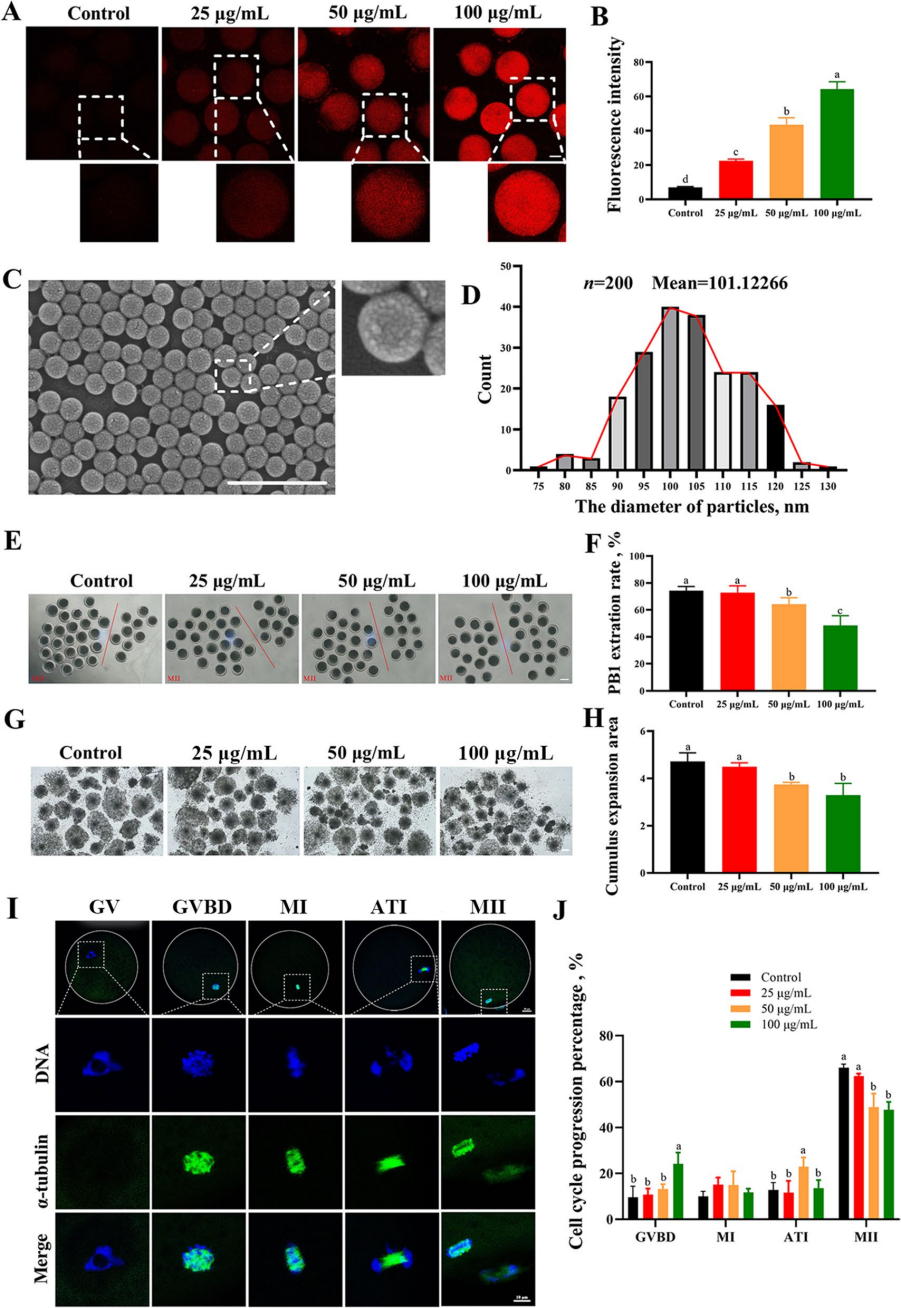
Effects of PS-NPs exposure on porcine oocyte maturation. **A **Images depicting red fluorescent-labelled polystyrene nanoplastics (PS-NPs) in oocytes. Red: PS-NPs. Scale bar: 50 μm. **B** Quantitative analysis of the PS-NPs fluorescence intensity in oocytes. *n* = 60. **C** The image of PS-NPs. Scale bar: 500 nm. **D** The range of PS-NPs size distribution. **E** Representative images of the first polar body (PB1) extrusion after 44 h of culture in vitro. The oocytes to the left of the red line have extruded the first polar body, while those to the right have not. Scale bar: 100 μm. **F** The PB1 extrusion rate in the control, 25 μg/mL, 50 μg/mL, and 100 μg/mL PS-NPs treated groups. *n* = 105. **G** Representative images of cumulus extrusion after 44 h of culture in vitro. Scale bar: 200 μm. **H** Cumulus cell expansion area in different groups. **I** Representative spindle morphology and chromosome alignment images in porcine oocytes during meiotic progression. Green: α-tubulin, blue: chromosome. GV, germinal vesicle; GVBD, germinal vesicle breakdown; MI, metaphase I; ATI, anaphase-telophase I; MII, metaphase II. **J** PS-NPs treatment disrupted cell cycle progression in porcine oocytes. *n* = 60. The letter “*n*” indicated the total number of oocytes in each group of three independent replicates. ^a−d^Values with different superscripts indicate statistical significance (*P* < 0.05)

Red fluorescent-labeled PS-NPs beads with a size of 100 nm were used to investigate whether PS-NPs can enter oocytes through membrane osmosis. As shown in Fig. 1A and B, PS-NPs osmosed into oocytes in a dose-dependent manner. Next, we evaluated the damaging effects of unmodified PS-NPs beads with size of 100 nm on porcine oocytes (Fig. 1C and D). The PB1 extrusion and cumulus expansion area were gradually inhibited as the PS-NPs concentration increased (Fig. 1E–H, *P* < 0.05). Next, the cell cycle progression of the PS-NPs exposed oocytes was analyzed. The characteristics of chromosomes and α-tubulin at different meiotic stages of porcine oocytes are shown in Fig. 1I, and the effects of PS-NPs on cell cycle progression were analyzed on this basis. The ratio of oocytes arrested at anaphase-telophase I (ATI) stage was markedly increased in the 50 μg/mL PS-NPs-treated group compared to that observed in the control group (Fig. 1J, 12.84% ± 1.61% and 22.91% ± 2.04%, respectively; *P* < 0.05) and that arrested at germinal vesicle breakdown (GVBD) stage was markedly increased in the 100 μg/mL PS-NPs treated group compared to the control group (9.62% ± 2.39% and 24.25% ± 2.41%, respectively; *P* < 0.05).

### PS-NPs caused cell cytoskeleton defects and DNA damage in porcine oocytes

**Fig. 2:**
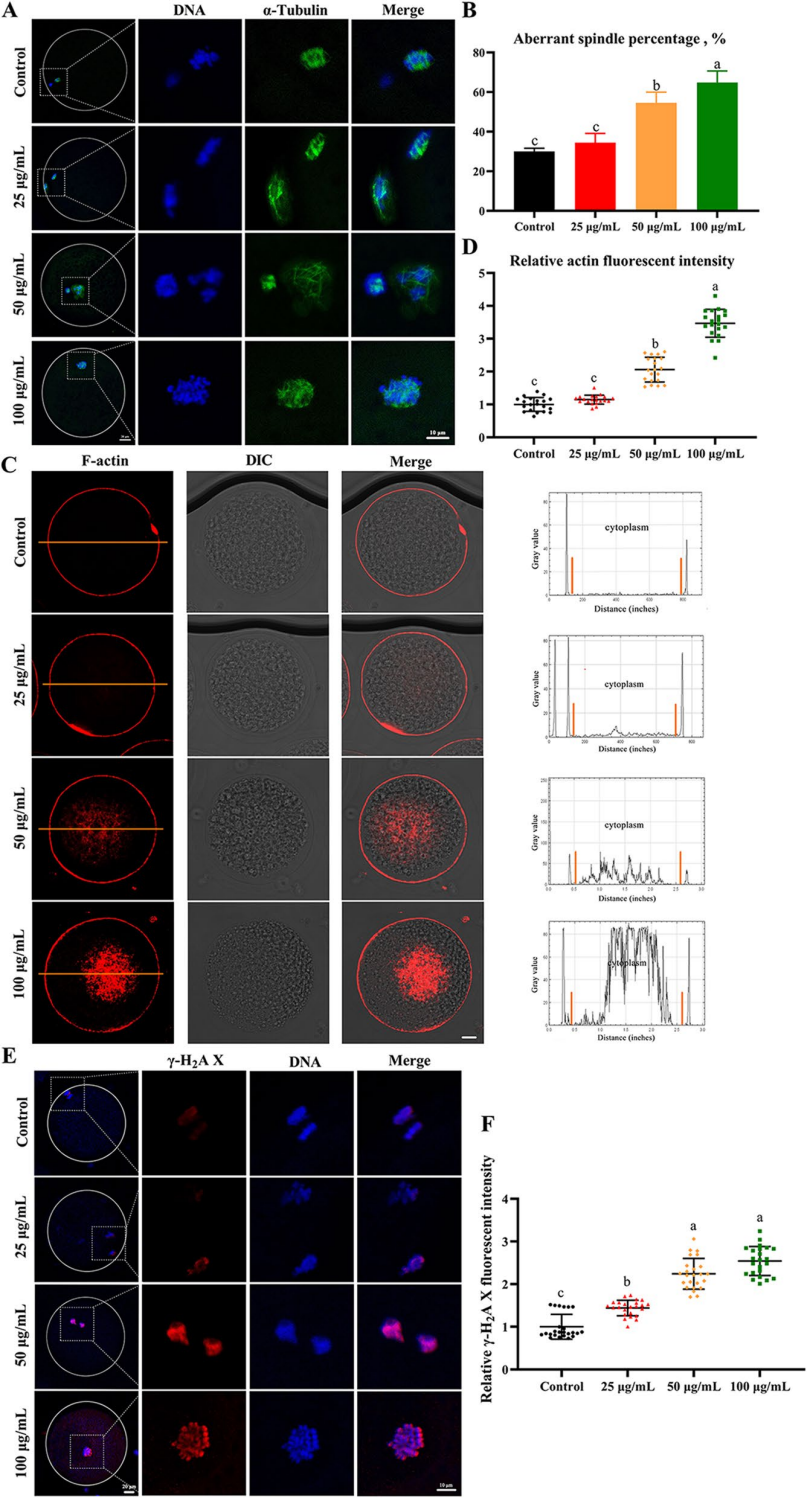
Effects of PS-NPs exposure on cell cycle progression and spindle morphology. **A** Representative images of spindle morphology and chromosome alignment in different groups after 44 h of culture. Green: α-tubulin, blue: chromosome. **B** Percentages of oocytes with aberrant spindles in different groups. *n* = 105. **C** Representative images of actin distribution in different groups after 44 h of culture. Red: F-actin. Scale bar: 20 μm. **D** Quantitative analysis of the F-actin fluorescence intensity in different groups of oocytes. *n* = 60. **E** Typical γ-H_2_A X images of porcine oocytes in different groups. Red: γ-H_2_A X, blue: chromosome. **F** Relative fluorescence intensity of γ-H_2_A X in different groups. *n* = 60. ^a−c^Values with different superscripts indicate statistical significance (*P* < 0.05)

The nuclear maturation of oocytes requires proper cytoskeletal assembly. In the current study, a laser scanning confocal microscope was used to assess whether PS-NPs had a negative impact on spindle morphologies and actin distribution. The control group exhibited a typical barrel-shaped spindle and a polar body in the cortex after 44 h of culture. However, in the 50 μg/mL-treated group, the oocytes showed aberrant α-tubulin and misaligned chromosomes (Fig. 2A). The incidence of abnormal spindle assembly increased from 30.00% ± 0.80% in the control group to 54.54% ± 2.72% in the 50 μg/mL PS-NPs-treated group (Fig. 2B, *P* < 0.05). The number of actin filaments in the cytoplasm was markedly increased in the 50 μg/mL PS-NPs-treated group than in the control group (Fig. 2C and D, 30.53 ± 5.58 and 14.83 ± 3.12, respectively; *P* < 0.05), indicating that the PS-NPs had adverse effects on the cell cytoskeleton. The toxicity of PS-NPs on DNA was also tracked by measuring the fluorescence intensity of γ-H_2_A X (Fig. 2E and F). The signals of γ-H_2_A X in the 25–100 μg/mL PS-NPs-treated groups were markedly elevated compared with the control group (*P* < 0.05).

### PS-NPs impaired the function and dynamics of mitochondria in porcine oocytes

**Fig. 3:**
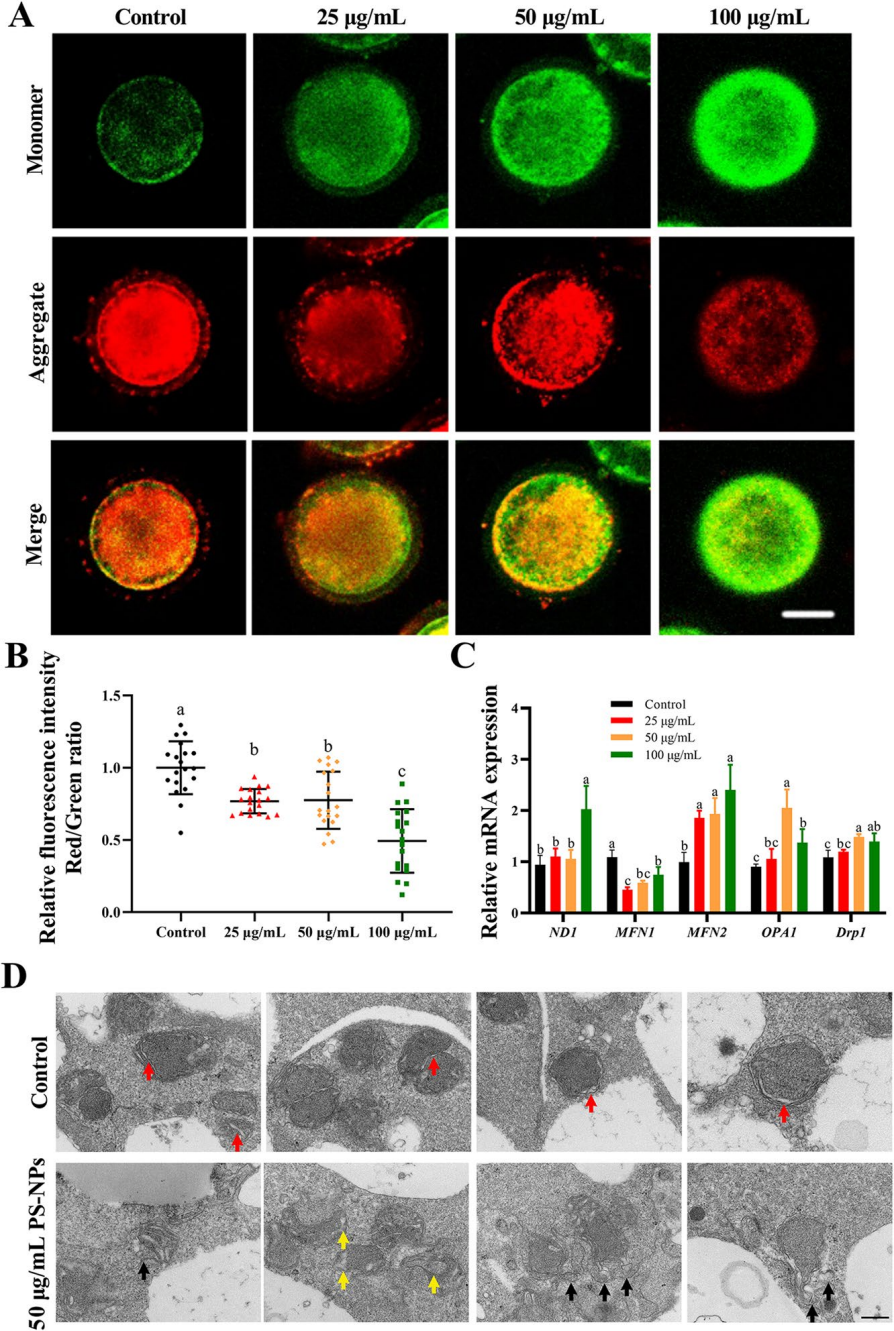
Effects of PS-NPs exposure on mitochondrial stress. **A** Representative images of JC-1 staining in different groups. Scale bar: 50 μm. **B** Quantitative analysis of the JC-1 red/green fluorescence intensity ratio in different groups. *n* = 60. **C** qPCR analysis of mtDNA and mitochondrial dynamic-related genes in different groups. **D** Ultrastructure observation of porcine oocyte mitochondria in different groups. Red arrows: mitochondrial cristae; black arrows: abnormal structure of mitochondrial membrane; yellow arrows: mitochondrial cristae broadening. Scale bar: 3 μm. ^a−c^Values with different superscripts indicate statistical significance (*P* < 0.05)

As shown in Fig. 3A, B and S2A, the MMP of oocytes in 25–100 μg/mL PS-NPs-treated groups was significantly lower than that in the control group (*P* < 0.05). Relative mtDNA copy numbers (the expression of NADH dehydrogenase submit 1, *ND1*) were markedly increased in 100 μg/mL PS-NPs treated group compared to the control group (Fig. 3C, *P* < 0.05). The expression of mitochondrial dynamics-related genes dynamin-related protein 1 (*Drp1*), mitofusin 2 (*MFN2*), and optic atrophy 1 (*OPA1*) was upregulated, while mitofusin 1 (*MFN1*) was downregulated in PS-NPs treatment groups (Fig. 3C, *P* < 0.05). Subsequently, the results of TEM showed that oocytes in the control group exhibited normal morphology with clear structures of the mitochondrial cristae and double membranes. In addition, broadened mitochondrial cristae and disrupted mitochondrial membranes were observed in the 50 μg/mL PS-NPs treated oocytes (Fig. 3D).

### PS-NPs induced oxidative stress and LPO in porcine oocytes

**Fig. 4:**
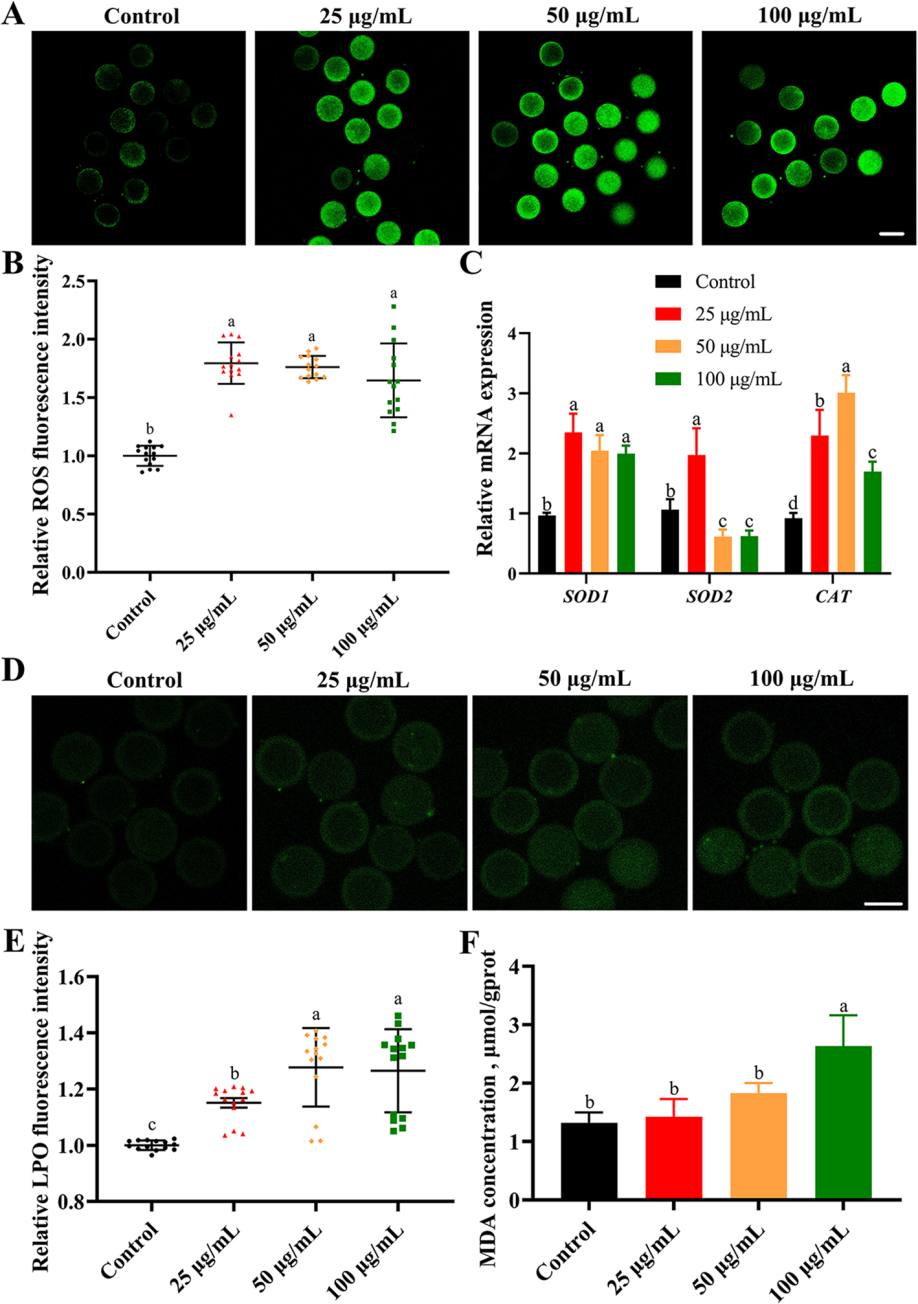
Effects of PS-NPs exposure on oxidative stress and lipid peroxidation **A** Representative images of ROS fluorescence signals in different groups. Green: DCFH-DA. Scale bar: 100 μm. **B** Relative fluorescence intensity of ROS in different groups. *n* = 60. **C** qPCR analysis of antioxidant-related genes in different groups. **D** Typical LPO images of porcine oocytes in different groups. Green: LPO. Scale bar: 50 μm. **E** Relative fluorescence intensity of LPO in different groups. *n* = 60. **F** MDA concentration of oocytes in different groups after 44 h of culture in vitro. ^a−d^Values with different superscripts indicate statistical significance (*P* < 0.05)

The fluorescence intensity of ROS in the PS-NPs-treated groups was markedly increased compared to the control group (Fig. 4A and B, *P* < 0.05). In addition, the PS-NPs treatment disrupted the mRNA expression of *SOD1*, *SOD2* and *CAT* (Fig. 4C, *P* < 0.05), indicating PS-NPs caused oxidative stress in porcine oocytes. Lipid peroxides were observably accumulated in oocytes after PS-NPs exposure (Fig. 4D and E, *P* < 0.05). MDA levels were markedly increased in the 100 μg/mL PS-NPs-treated group than in the control group (Fig. 4F, *P* < 0.05).

### PS-NPs exposure led to ferroptosis in porcine oocytes

**Fig. 5:**
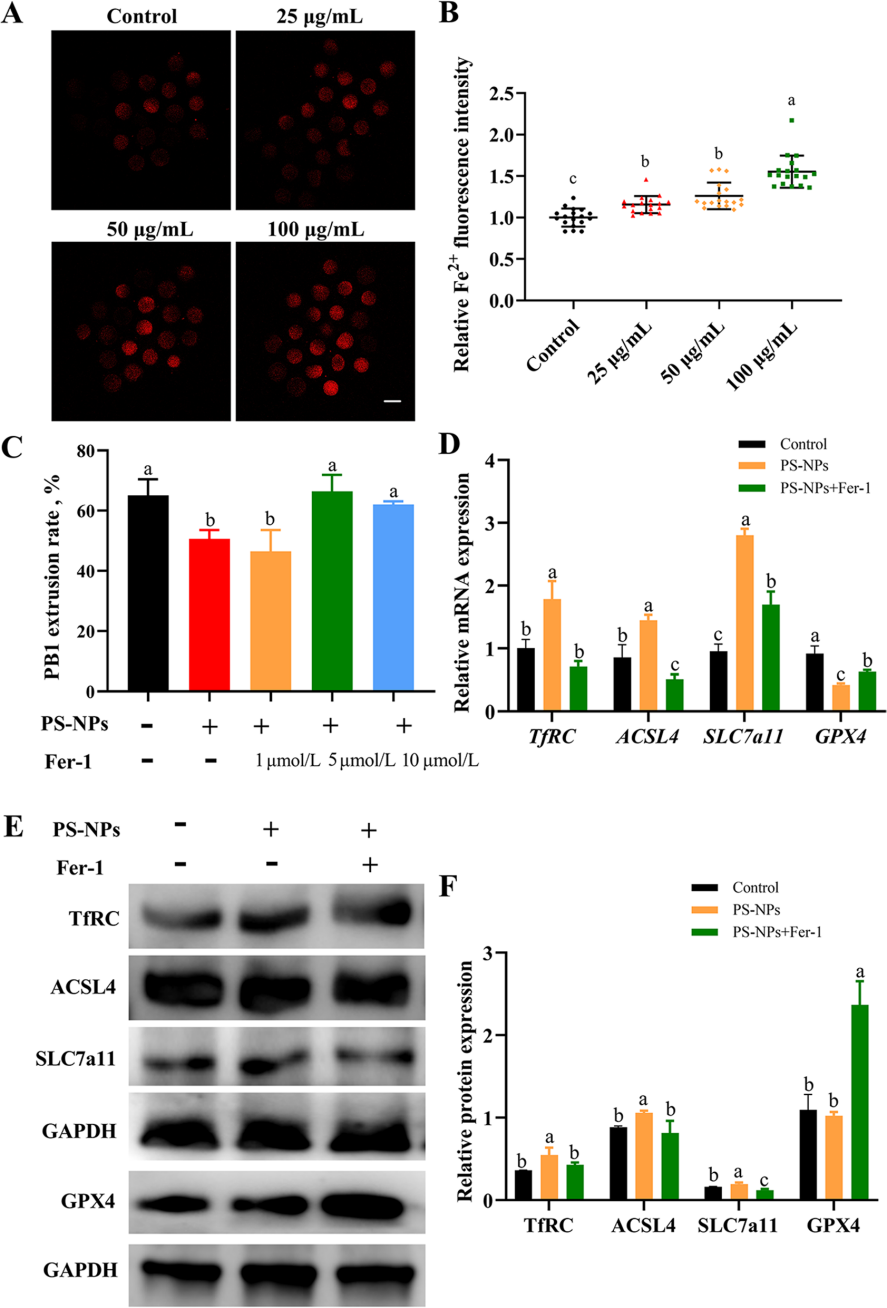
Effects of PS-NPs exposure on the ferroptosisof porcine oocytes.** A** Representative images of Fe^2+^ fluorescence signals in different groups. Red: Fe^2+^. Scale bar: 100 μm. **B** Relative fluorescence intensity of Fe^2+^ in different groups. *n* = 60. **C** Effects of gradient concentrations of ferrostatin-1 (Fer-1) on PB1 extrusion rate of the 50 μg/mL PS-NPs treated oocytes. *n* = 105. **D** qPCR analysis of ferroptosis-related genes in the control, 50 μg/mL PS-NPs, 50 μg/mL PS-NPs + 5 μmol/LM Fer-1 cotreatment group. **E** and **F** The protein expression of TfRC (85 kDa), ACSL4 (79 kDa), SLC7a11 (55 kDa) and GPX4 (22 kDa) in the control, PS-NPs and PS-NPs + Fer-1 cotreatment group. ^a−c^Values with different superscripts indicate statistical significance (*P* < 0.05)

The fluorescence intensity of Fe^2+^ signals in the 25–100 μg/mL PS-NPs-treated groups was markedly elevated compared to that observed in the control group (Fig. 5A and B, *P* < 0.05). The concentration of 50 μg/mL PS-NPs was used in the subsequent experiments. Fer-1 was cotreated with PS-NPs as ferroptosis-inhibited group to investigate whether the toxic effects of PS-NPs on porcine oocytes were mediated by ferroptosis. Fer-1 (5 and 10 μmol/L) treatment prominently reversed the inhibition of PB1 extrusion induced by PS-NPs (*P* < 0.05) (Fig. 5C and S1A). However, when oocytes were treated with Fer-1 (5 μmol/L) alone, there was no significant change in PB1 extrusion (Fig. S1B and C, *P* > 0.05). According to the results, a concentration of 5 μmol/L Fer-1 was selected for the subsequent experiments. The mRNA expression of *TfRC, ACSL4* and *SLC7a11* was upregulated and *GPX4* was downregulated after PS-NPs treatment (Fig. 5D, *P* < 0.05), likewise, the results of Western blot showed that PS-NPs exposure led to an increase in TfRC, ACSL4, and SLC7a11 protein expression (Fig. 5E and F, *P* < 0.05). However, the effect of PS-NPs on the expression of genes and proteins related to ferroptosis was reversed after Fer-1 cotreatment.

**Fig. 6:**
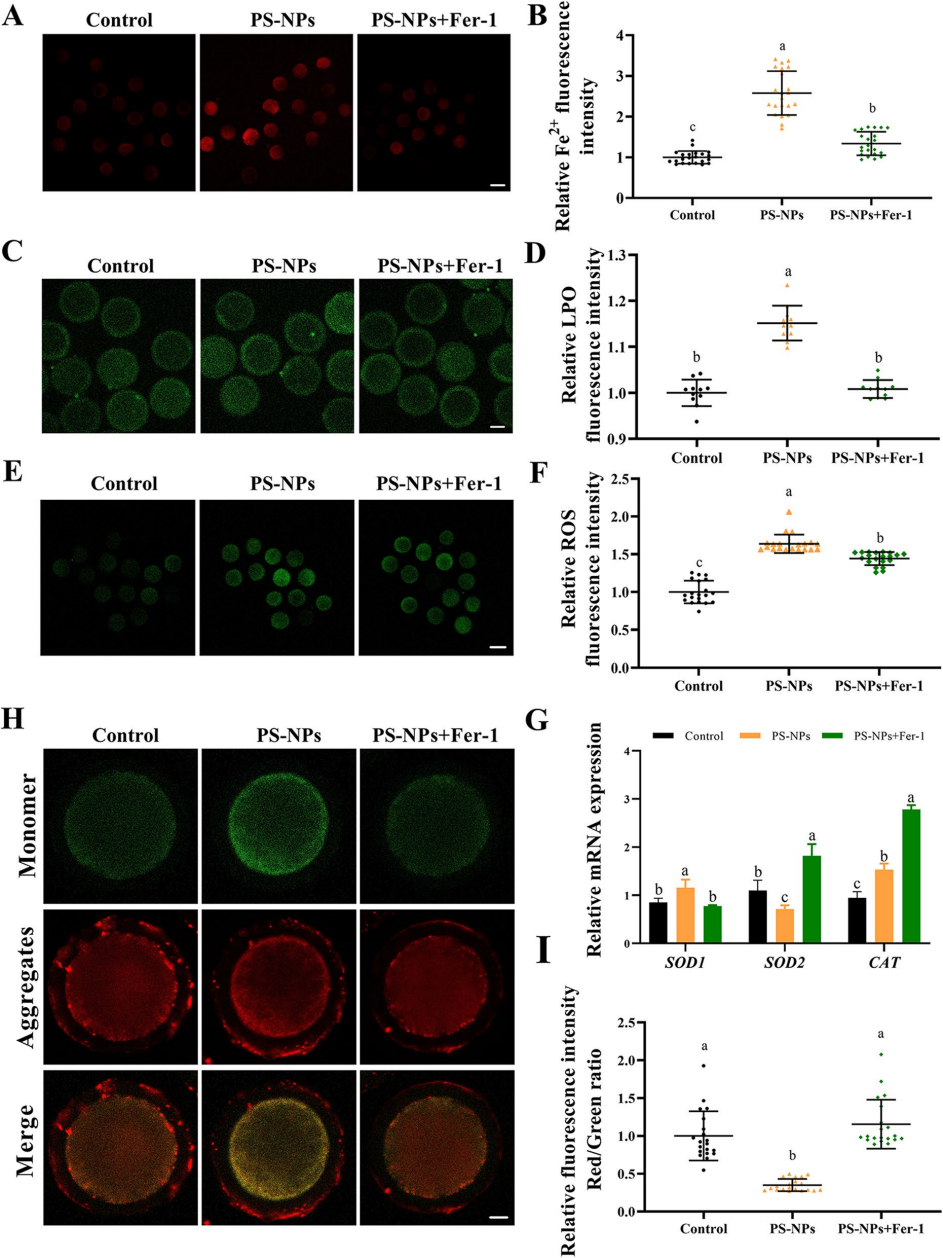
Ferroptosis inhibition ameliorated oxidative stress and mitochondrial stress in PS-NPs exposed oocytes. **A** Representative images of Fe^2+^ fluorescence signals in the control, PS-NPs and PS-NPs + 5 μmol/L Fer-1 cotreatment group. Red: Fe^2+^. Scale bar: 100 μm. **B** Relative fluorescence intensity of Fe^2+^ in different groups. *n* = 60. **C** Typical LPO images of porcine oocytes in different groups. Green: LPO. Scale bar: 50 μm. **D** Relative fluorescence intensity of LPO in different groups. *n* = 60. **E** Representative images of ROS fluorescence signals in the control, PS-NPs, PS-NPs + Fer-1 cotreatment group. Green: DCFH-DA. Scale bar: 100 μm. **F** Relative fluorescence intensity of ROS in different groups. *n* = 60. **G** qPCR analysis of antioxidant-related genes in the control, PS-NPs and PS-NPs + Fer-1 cotreatment group. **H** Representative images of JC-1 staining in the control, PS-NPs + Fer-1 cotreatment group. Scale bar: 50 μm. **I** Quantitative analysis of the ratio of JC-1 red/green fluorescence intensity in different groups. *n* = 60. ^a−c^Values with different superscripts indicate statistical significance (*P* < 0.05)

**Fig. 7:**
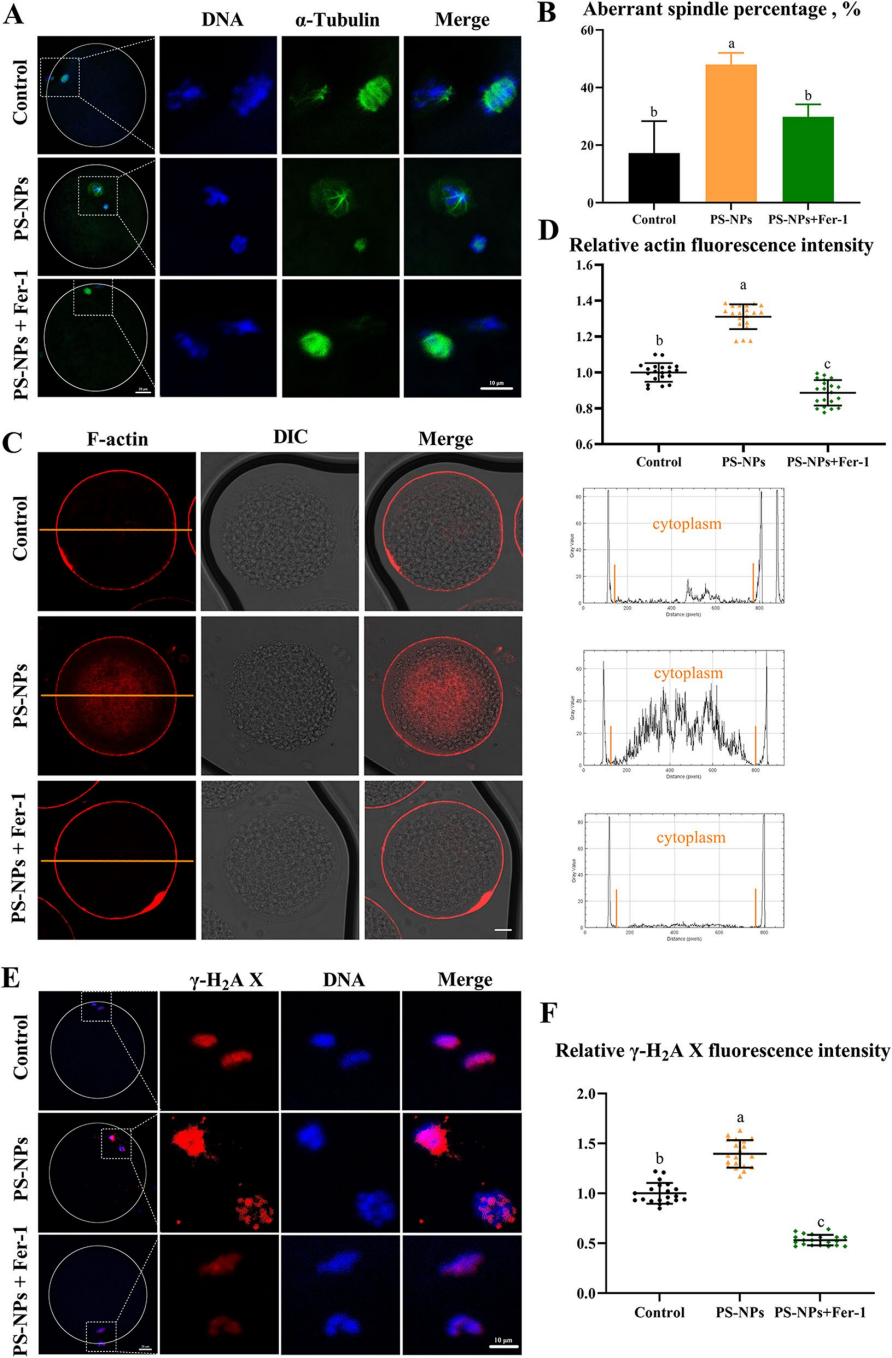
Ferroptosis inhibition alleviated the defects of cytoskeleton in PS-NPs exposed oocytes. **A** Representative images of spindle morphology and chromosome alignment in the control, PS-NPs and PS-NPs + Fer-1 cotreatment group. Green: α-tubulin, blue: chromosome. **B** Percentages of oocytes with aberrant spindles in different groups. *n* = 105. **C** Representative images of actin distribution in the control, PS-NPs and PS-NPs + Fer-1 cotreatment group. Red: F-actin. Scale bar: 20 μm. **D** Quantitative analysis of the F-actin fluorescence intensity in the oocytes of different groups. *n* = 60. **E** Typical γ-H_2_A X images of porcine oocytes in the control, PS-NPs and PS-NPs + Fer-1 cotreatment group. Red: γ-H_2_A X, blue: chromosome. **F** Relative fluorescence intensity of γ-H_2_A X in different groups. *n* = 60. ^a−c^Values with different superscripts indicate statistical significance (*P* < 0.05)

To verify the toxic mechanism of PS-NPs, we explored whether the inhibition of ferroptosis can alleviate the toxic effects on porcine oocytes. As shown in Fig. 6A–F, cotreatment of Fer-1 reduced the accumulation of Fe^2+^ (*P* < 0.05), LPO (*P* < 0.05) and ROS (*P* < 0.05) compared to PS-NPs group. Meanwhile, the disruption of antioxidant enzymes-related genes was effectively alleviated by Fer-1. (Fig. 6G, *P* < 0.05). PS-NPs + Fer-1 cotreatment significantly increased the MMP in PS-NPs-exposed oocytes (Fig. 6H, I and S2B, *P* < 0.05). Moreover, the percentage of abnormal spindle (Fig. 7A and B, *P* < 0.05), actin intensity in the cytoplasm (Fig. 7C and D, PS-NPs vs. PS-NPs + 5 μmol/L Fer-1: 59.36 ± 3.13 vs. 40.13 ± 3.21, *P* < 0.05) and the expression of γ-H_2_A X (Fig. 7E and F, PS-NPs vs. PS-NPs + 5 μmol/L Fer-1: 13.40 ± 1.32 vs. 5.10 ± 0.51, *P* < 0.05) were markedly decreased in PS-NPs + Fer-1 cotreatment group compared to PS-NPs group.

## Discussion

The threat of PS-NPs accumulating in different tissues and cells has attracted increasing attention. Our data elucidate that PS-NPs could enter oocytes, cause mitochondrial dysfunction and oxidative stress, and induce lipid peroxidation and ferroptosis, eventually resulting in the failure of oocyte maturation.

NPs can enter the cell membrane through two main pathways: active cell endocytosis and passive membrane penetration [24]. When NPs pass through the cell membrane, they can induce mechanical damage to the lipid bilayer and membrane transporters [25]. The smaller NPs could be easily taken up via endocytosis [26]. In the current study, PS-NPs diffused through oocytes and subsequently disrupted the meiotic progression and oocyte maturation stages. PS-NPs exposure compromised the subcellular structure of oocyte cytoskeleton, leading to abnormal spindle segregation and actin diffusion. A prior study revealed that PS-NPs could exert negative effects on cytoplasmic microtubules and microfilaments [10]. F-actin is regarded as the sole cytoskeletal regulator of active spindle migration toward the cortical side [27–29]. In the present study, our assumption was that PS-NPs interact with microfilaments and inhibit actin polymerization, thus influencing spindle migration during anaphase of meiotic division. PS-NPs, at high concentration, could cause cytotoxicity of NPs via disrupting vital cellular surface structures [10]. In this study, PS-NPs at a dose of 100 μg/mL induced maturation oocyte arrest at GVBD stage, which could be attributable to the high cytotoxicity at prophase of the first meiotic prophase.

The reproductive systems of animals are well known to be seriously threatened by DNA damage, as evidenced by atresia and impaired follicular growth, necroptosis of ovarian granulosa cells, and irreversible damage to testicular tissue [30–32]. DNA damage in mammalian oocytes and female germ cells can result in genetic abnormalities in the growing embryo [33]. DNA double-strand breaks are recognized by the presence of the phosphorylated form of H_2_A X, γ-H_2_A X [34]. Our research revealed that exposure to PS-NPs increased the expression of γ-H_2_A X, indicating that PS-NPs may have a detrimental effect on DNA.

Mitochondria are cellular powerhouses in eukaryotes that continuously supply energy to cells. External stimuli can disrupt the steady state of mitochondria and initiate mitochondria stress [35]. ND1, which is the specific rimer for the coding region of mtDNA, is determined to represent relative mtDNA copy number [36]. Our results confirmed that PS-NPs could compromise mitochondrial homeostasis, including membrane depolarization, DNA damage in mitochondria, disruption of mitochondrial dynamics and abnormal morphology. We speculated that PS-NPs can interact with mitochondrial and further affect the fission and fusion of mitochondria. MFN1, MFN2 and OPA1 are key regulators of mitochondrial fusion and Drp1 is a primary regulator of mitochondrial fission [35, 37]. In this study, oocytes in the PS-NPs treatment group showed abnormalities in mitochondrial DNA synthesis, MMP decreased and disorder of mitochondrial fusion and fission, indicated that mitochondrial stress and dynamic abnormal. Once this dynamic balance is broken, the mitochondria may show functional defects, which are closely related to an increase of ROS synthesis [38]. It has been reported that ROS oversynthesis can further lead to mitochondrial dynamics abnormal [39]. Normal oocytes maturation requires a low concentration of ROS, while exposure to higher concentrations of ROS causes damage to cellular macromolecules and organelle, eventually lead to the failure of meiosis [40, 41]. It has been confirmed that extreme ROS generated by oxidative phosphorylation is the main toxic effect of PS particles in many tissues and cells, such as the ovary, intestinal barrier and N_2_A cells [11, 42, 43]. In this study, PS-NPs exposure led to mitochondrial stress in oocytes, causing excessive ROS generation. High levels of ROS further induce damage to oocyte organelles, resulting in the failure of oocyte maturation.

Overexpressed ROS will attack PUFA and then caused LPO [44, 45]. LPO is the oxidative deterioration of lipids or PUFAs, which is essentially a chain reaction of free radicals caused by oxidative stress [46]. PS particles have been shown to cause LPO in carbs, mice and animal cells [47, 48]. We investigated the effects of PS-NPs on LPO in porcine oocytes and found that the MDA content was markedly increased in the PS-NPs treatment group. Ferroptosis is characterized by iron overload-dependent accumulation of ROS and LPO to lethal levels [18]. We also found that PS-NPs substantially increased intracellular Fe^2+^ levels. Previous studies have confirmed that nanoparticles can induce ferroptosis by disrupting iron metabolism [49, 50]. Tang et al. [21] have demonstrated that PS-NPs exposure during pregnancy causes ferroptosis in the small intestine. In that way, we investigated how exposure to PS-NPs during meiosis affected the expression of ferroptosis associated genes and proteins in porcine oocytes. We characterized the toxicity mechanism of PS-NPs on porcine oocytes by examining four ferroptosis-related genes (*TfRC, ACSL4, GPX4* and *SLC7a11*). GPX4 is a crucial protein that protects against ferroptosis by inhibiting phospholipid hydroperoxides and hence reducing the level of lipoxygenase-mediated LPO [45]. SLC7a11 is also a negatively regulated protein of ferroptosis whose expression abrogates cellular redox homeostasis [44]. ACSL4 is important for the production of PUFAs, which are required for the activation of ferroptosis. TfRC mediated endocytosis deliver iron to different tissues through transferrin. Excess intracellular iron may drive LPO under ferroptotic conditions [51]. In the present study of porcine oocytes, we found that the expression of TfRC, ACSL4 and SLC7a11 increased significantly in porcine oocytes. These data indicated that PS-NPs caused ferroptosis by upregulating the expression of TfRC, SLC7a11 and ACSL4.

Ferroptosis is a regulated cell death that depends on iron and ROS [52]. We further explored whether the inhibition of ferroptosis could alleviate the toxicity of PS-NPs to oocytes. Fer-1, a specific small-molecule inhibitor of ferroptosis [44], which can prevent LPO through the activity of radical-trapping antioxidants to subvert ferroptosis [53]. A recent study revealed that Fer-1 treatment could decrease ROS and MDA content, inhibit LPO and enhance the antioxidant capacity of spermatogenic cells [54], which is consistent with the findings observed in porcine oocytes. The results showed that Fer-1 treatment also increased antioxidant activity and restored mitochondrial stress and dysfunctional cytoskeletal dynamics.

## Conclusion

The results of the present study showed for the first time that ferroptosis occurs in PS-NPs-exposed porcine oocytes and has significant toxic effects on the cytoskeleton and mitochondria. The underlying mechanism may be that increased ROS and MDA content leads to ferroptosis via upregulation of TfRC and ACSL4. The findings of the present study contribute to estimating the risk of PS-NPs exposure affecting animal reproductive health.

### Supplementary Information

**Additional file 1**. Primer sequences used for quantitative PCR.

**Additional file 2:** **Fig. S1**. **A** Representative images of PB1 extrusion after 44 h of culture with different concentrations of Fer-1. Scale bar: 100 μm. **B** Representative images of PB1 extrusion in control group and 5 μmol/L Fer-1 treated group. Scale bar: 100 μm. *n *= 105. The letter “*n*” indicated the total number of oocytes in each group of 3 independent replicates. **C** The PB1 extrusion rate in Control and 5 μmol/L Fer-1 treated group. *n* = 105.

**Additional file 3: Fig. S2**. Quantitative analysis of JC-1 red and green fluorescence intensity in different groups. *n* = 60.

## Data Availability

Data will be made available on reasonable request.

## Abbreviations

4-HNE: 4-Hydroxynonenal
ACSL4: Acyl-CoA synthetase long-chain family member 4
ATI: Anaphase-telophase I
COCs: Cumulus oocyte complexes
Drp1: Dynamin-related protein 1
Fer-1: Ferrostatin-1
GPX4: Glutathione peroxidase 4
GVBD: Germinal vesicle breakdown
LPO: Lipid peroxidation
MDA: Malondialdehyde
MFN1: Mitofusin 1
MFN2: Mitofusin 2
MMP: Mitochondrial membrane potential
MPs: Microplastics
ND1: NADH dehydrogenase submit 1
OPA1: Optic atrophy 1
PS-NPs: Polystyrene nano-plastics
PUFA: Polyunsaturated fatty acid
qPCR: Quantitative PCR
ROS: Reactive oxygen species
SLC7a11: Solute carrier family 7 member 11
TEM: Transmission electron microscopy
TfRC: Transferrin receptor
PB1: The first polar body
