# Supplementary material for: Polystyrene nanoplastic exposure actives ferroptosis by oxidative stress-induced lipid peroxidation in porcine oocytes during maturation
Source: J Anim Sci Biotechnol. 2024 Sep 3;15:117. doi: 10.1186/s40104-024-01077-6 (PMC11370062; doi:10.1186/s40104-024-01077-6)
Supplement: Supplementary file 1 — Additional file 1. Primer sequences used for quantitative PCR. [file 40104_2024_1077_MOESM1_ESM.docx]

**Additional file 1** Primer sequences used for quantitative PCR

| **Gene** | **Primer sequence** | **Fragment size, bp** |
| --- | --- | --- |
| *ND1* | F-5'-TCCTACTGGCCGTAGCATTCCT-3' | 165 |
|  | F-5'-TTGAGGATGTGGCTGGTCGTAG-3' |  |
| *MFN1* | F-5'-CTTCGATGGGCATCATTGTTGTTGG-3' | 135 |
|  | F-5'-TCTCCTTGGCACGGGTGGTC-3' |  |
| *MFN2* | F-5'-GTTGTTGGAGGAGTGGTGTGGAAG-3' | 156 |
|  | F-5'-CTCGCTGGCGTACTCTACAAACTG-3' |  |
| *OPA1* | F-5'- GGACTACAGAGGATGGTGCTTGTTG-3' | 138 |
|  | F-5'-ACACAGTATGATGGCGTTGGGATTC-3' |  |
| *Drp1* | F-5'-CAGCAGAGGAGCAACCAGATGAAC-3' | 150 |
|  | F-5'-ATTCTCGGCGGTCAGCACAAAC-3' |  |
| *SOD1* | F-5'-ACCTGGGCAATGTGACTG-3' | 197 |
|  | R-5'-TCCAGCATTTCCCGTCT-3' |  |
| *SOD2* | F-5'-GGACAAATCTGAGCCCTAACG-3' | 184 |
|  | R-5'-CCTTGTTGAAACCGAGCC-3' |  |
| *CAT* | F-5'-AACTGTCCCTTCCGTGCTA-3' | 195 |
|  | R-5'-CCTGGGTGACATTATCTTCG-3' |  |
| *TfRC* | F-5'-TGGCTCGGCAGGTAGATGGTG-3' | 96 |
|  | F-5'-TGTGGTTACTCCTTGTGTTGCTGTC-3' |  |
| *ACSL4* | F-5'-GGAAGTCCATATCGCTCTGTCACAC-3' | 135 |
|  | F-5'-CTCCCTGGTCCCAAGGCTGTC-3' |  |
| *SLC7A11* | F-5'-TCTTTGTTGCCCTCTCCTGCTTTG-3' | 130 |
|  | F-5'-GTGTGTTTGCGGATGTGAATCATGG-3' |  |
| *GPX4* | F-5'-GCCTGTTCCGCCTGCTGAAG-3' | 146 |
|  | F-5'-CATGTGCCCGTCGATGTCCTTG-3' |  |
| *β-actin* | F-5'-CTGCGGCATCCACGAAACT-3' | 147 |
|  | F-5'-AGGGCCGTGATCTCCTTCTG-3' |  |
