# Supplementary figures and images for: Polystyrene nanoplastic exposure actives ferroptosis by oxidative stress-induced lipid peroxidation in porcine oocytes during maturation
Source: J Anim Sci Biotechnol. 2024 Sep 3;15:117. doi: 10.1186/s40104-024-01077-6 (PMC11370062; doi:10.1186/s40104-024-01077-6)

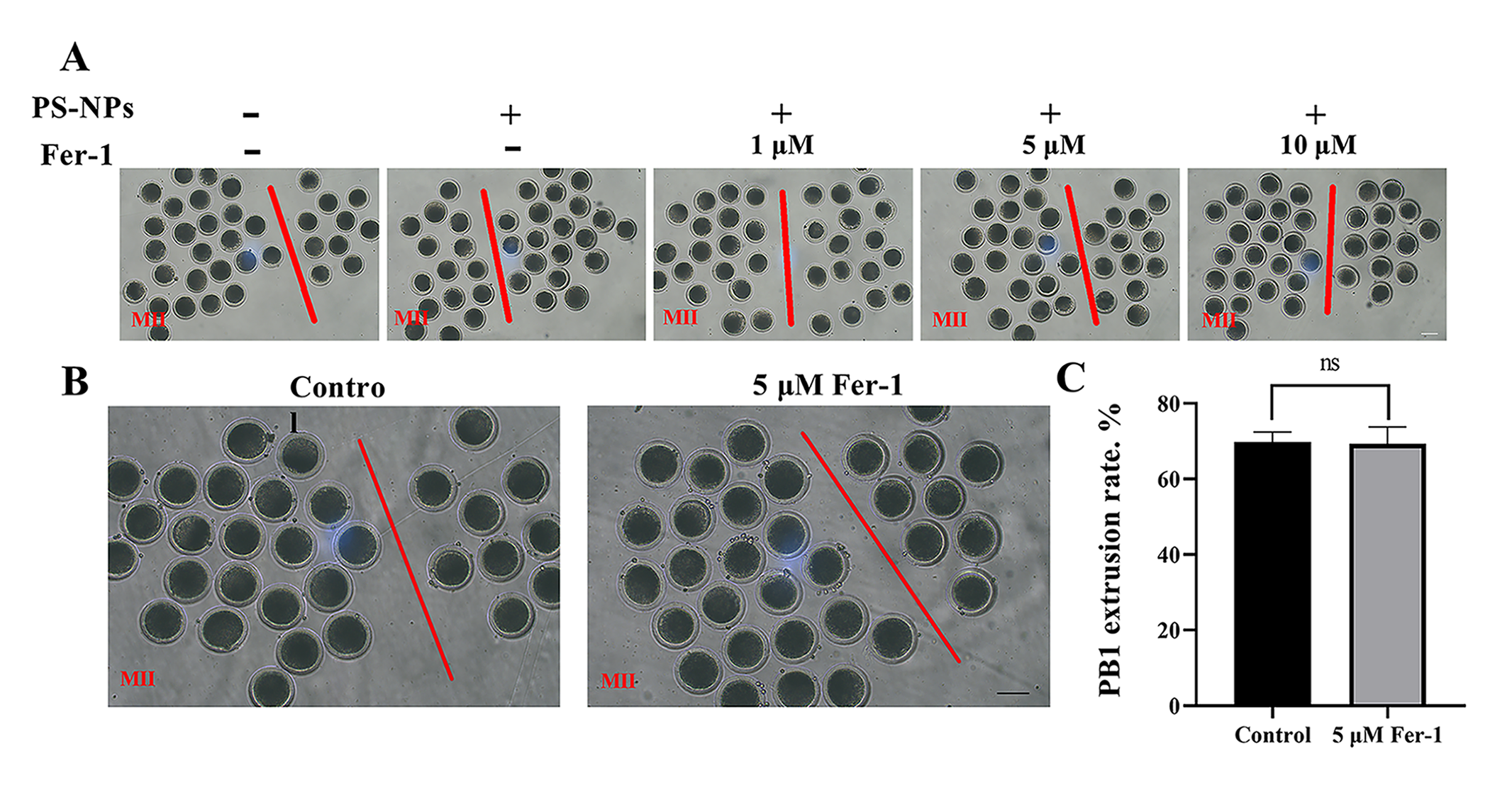

Supplement: Supplementary file 2 — Additional file 2: Fig. S1. A Representative images of PB1 extrusion after 44 h of culture with different concentrations of Fer-1. Scale bar: 100 μm. B Representative images of PB1 extrusion in control group and 5 μmol/L Fer-1 treated group. Scale bar: 100 μm. n = 105. The letter “n” indicated the total number of oocytes in each group of 3 independent replicates. C The PB1 extrusion rate in Control and 5 μmol/L Fer-1 treated group. n = 105. [file 40104_2024_1077_MOESM2_ESM.tif]

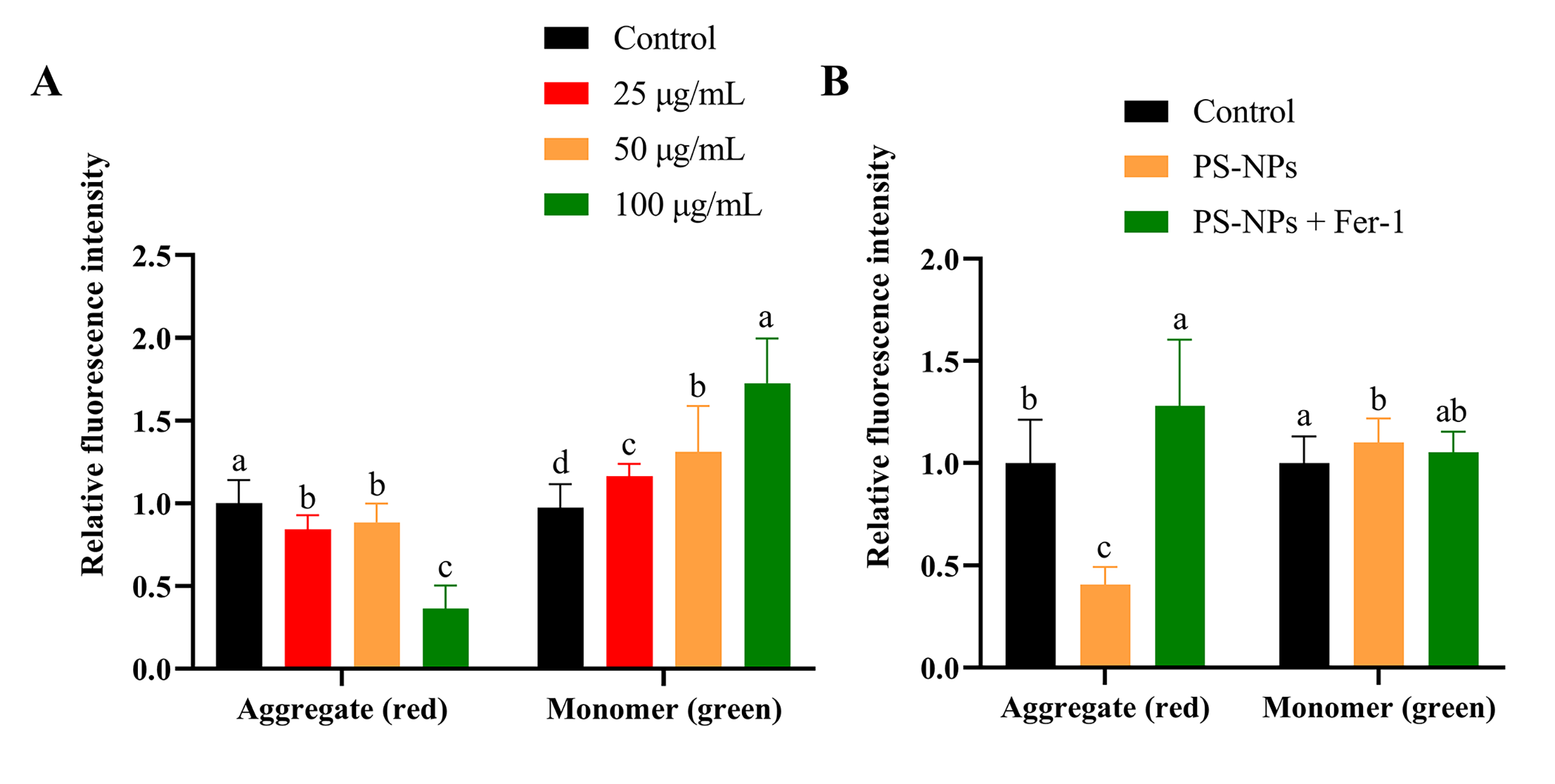

Supplement: Supplementary file 3 — Additional file 3: Fig. S2. Quantitative analysis of JC-1 red and green fluorescence intensity in different groups. n = 60. [file 40104_2024_1077_MOESM3_ESM.tif]
